# Prevalence and risk factors associated with infection of major diarrhoegenic protozoan parasites in HIV patients with ART at Silchar Medical College and Hospital, Assam, India

**DOI:** 10.1186/1471-2334-14-S3-P4

**Published:** 2014-05-27

**Authors:** Joyobrato Nath, Gulzar Hussain, Sankar K Ghosh, Prithwiraj Bhattacharjee, Baby Singha

**Affiliations:** 1 Department of Zoology, Gurucharan College, Silchar, Assam, India; 2 Department of Biotechnology, Assam University, Silchar, Assam, India; 3 Department of Medicine, Silchar Medical College and Hospital, Silchar, Assam, India

## Background

In developing countries diarrhoegenic Protozoan parasites mainly *Cryptosporidium parvum*, *Enterocytozoon bieneusi*, *Giardia duodenalis* and *Entamoeba histolytica* have been implicated as major contributors to morbidity and mortality in HIV patients.

## Methods

In this cross sectional study, 274 stool samples collected from HIV patients with ART at SMCH, Silchar, Assam, India were initially examined for cysts/oocysts stages using iodine and modified ZN staining techniques respectively and subsequently confirmed through PCR assay using specific primers. Data on associated risk factors were obtained by interviewing all 274 patients with ART.

## Results

The overall prevalence of the four diarrhoegenic Protozoan parasitic infections was estimated to be 22.9% (95% CI= 18.40, 28.33) which included *E. bieneusi* was the most common (12.4%; 95% CI= 9.02, 16.84) followed by *C. parvum* (9.5%; 95% CI= 6.56, 13.54), *E. histolytica* (8.1%; 95% CI= 5.36, 11.86), and *G. duodenalis* (4.7%; 95% CI= 2.79, 7.94). The overall infection in patients with CD4 count ≤200 cells/µL was significantly higher as compared with patients with CD4 counts 200-300 and ≥ 300 cells/ µL (*p* < 0.001). Logistic regression analysis showed close animal contact (OR= 2.42; 95% CI= 1.36, 4.29), river or well as water source (OR= 5.97; 95% CI= 3.22, 11.05), unhygienic toilet (OR= 3.43; 95% CI= 1.91, 6.17) and poor living condition (OR= 2.12; 95% CI= 1.19, 3.74) significantly increase the likelihood of infection (*p*< 0.01).

## Conclusion

The study emphasizes the need for routine screening of diarrhoegenic Protozoan parasite in HIV patients along with well education to patients about practicing personal hygiene.

